# Perforation of the Nasal Septum in a Colorectal Cancer Patient Treated With Aflibercept: A Case Report

**DOI:** 10.7759/cureus.26780

**Published:** 2022-07-12

**Authors:** Javier David Benitez Fuentes, Alfonso Lopez de Sa Lorenzo, Alberto Elpidio Calvo Elias, Carmen Toledano Rojas, Monica Granja Ortega

**Affiliations:** 1 Health Research Institute of the Hospital Clínico San Carlos (IDISSC), Hospital Clínico San Carlos, Madrid, ESP; 2 Medical Oncology, Hospital Clinico San Carlos, Madrid, ESP; 3 Internal Medicine, Hospital Clinico San Carlos, Madrid, ESP

**Keywords:** skin and mucosal toxicity, a case report, antiangiogenic drug, adverse event, nasal septum perforation, colorectal cancer, aflibercept

## Abstract

Aflibercept is an antiangiogenic agent used in patients with metastatic colorectal cancer who have progressed to a first-line oxaliplatin-based regimen. The main adverse effects (AEs) of antiangiogenic agents are fatigue, asthenia, anorexia, hypertension, proteinuria, urinary tract infection, diarrhea, and neutropenia. Other AEs, such as hemorrhage, thromboembolic events, and gastrointestinal perforation, are much less frequent. Nasal septal perforation caused by antiangiogenic agents is even rarer. The published literature on this subject is scarce. Here, we report the case of a 54-year-old male with metastatic colorectal cancer undergoing treatment with leucovorin, fluorouracil (5-FU), irinotecan, and aflibercept who presented with epistaxis and nasal congestion. An otolaryngologist performed a rhinoscopy that revealed a perforation of the nasal septum. Aflibercept was withdrawn first, and local treatment was applied with lubricant and antibacterial lotions. It was considered a non-life-threatening side effect, and given the high risk of not continuing treatment in this patient with a recent recurrence, aflibercept was reintroduced in combination with leucovorin, 5-FU, and irinotecan. The patient continued local treatment and follow-up with medical oncology and otolaryngology with gradual improvement of symptoms. Follow-up was discontinued due to disease progression and death after 16 months of the event.

## Introduction

Aflibercept is a recombinant fusion protein formed by portions of the vascular endothelial growth factor (VEGF) binding sites to the extracellular domains of human VEGF receptors 1 and 2, fused to an Fc portion of human immunoglobulin G1 (IgG1) [1]. VEGF is an essential regulator of angiogenesis, primarily activated by hypoxia, and overexpressed in several malignancies, including colorectal cancer. VEGF plays an important role in tumor vascularity, proliferation, progression, invasion, and metastasis [2]. Aflibercept performs its function by binding to various angiogenic molecules, preventing their binding to their native receptors of the VEGF family and thus acting as an antiangiogenic agent. It is indicated, in combination with FOLFIRI (leucovorin, 5-FU, irinotecan), in patients with metastatic colorectal cancer who have progressed to a first-line oxaliplatin-based regimen [1].

Regarding the most frequent adverse events (AEs) related to antiangiogenic drugs, we find fatigue, asthenia, anorexia, hypertension, proteinuria, urinary tract infection, diarrhea, and neutropenia. Also described but less frequent, there are thromboembolic and hemorrhagic events as well as fistula in any location [3]. In the published literature on the use of antiangiogenic drugs, it has been found that perforation of the nasal septum can also occur, the compound with the most data regarding this is bevacizumab [4-5]. It has been hypothesized that this AE could be due to the nasal cartilage being poorly vascularized, in addition to the argument that these drugs may further compromise the sparse vasculature of this tissue [4].

Here we present the case of a 54-year-old man diagnosed with colorectal cancer stage IV treated with FOLFIRI (leucovorin calcium (calcium folinate), 5-fluorouracil, and irinotecan) and aflibercept who presented with nasal septum necrosis and perforation during the treatment.

## Case presentation

We describe the case of a 54-year-old Slavic male with no previous medical records. In January 2019, he was diagnosed with acute appendicitis. The patient underwent an urgent appendicectomy. Pathologists found no signs of malignancy in this first surgery. In March 2019, he was hospitalized with the chief complaint of abdominal pain associated with fever. A computed tomography (CT) scan revealed multiple intra-abdominal abscesses in the right iliac fossa. A second urgent surgery was performed. The surgical team detected an abscessed mass on the hepatic flexure of the colon with perforation to the retroperitoneum and abdominal cavity. The surgeons then performed a right hemicolectomy, drainage, and ileocolic anastomosis. Pathology showed a poorly differentiated enteroid adenocarcinoma pT4apN1c, stage IIIB, wild-type RAS, BRAF V600E mutation.

In May 2019, the patient started adjuvant therapy with capecitabine and oxaliplatin, withdrawing oxaliplatin after the third cycle due to moderate-severe nausea and diarrhea. He received seven cycles until October 2019. A follow-up CT in November 2019 showed disease recurrence in the right iliac fossa confirmed in a positron emission tomography CT in December 2019. The patient then started therapy with FOLFIRI plus aflibercept, reporting epistaxis and nasal congestion after five cycles. The subject had no previous history of nasal pathology, hypertension, or hemorrhagic diathesis. The Medical Oncology team then contacted the otolaryngology department for further evaluation.

**Figure 1:**
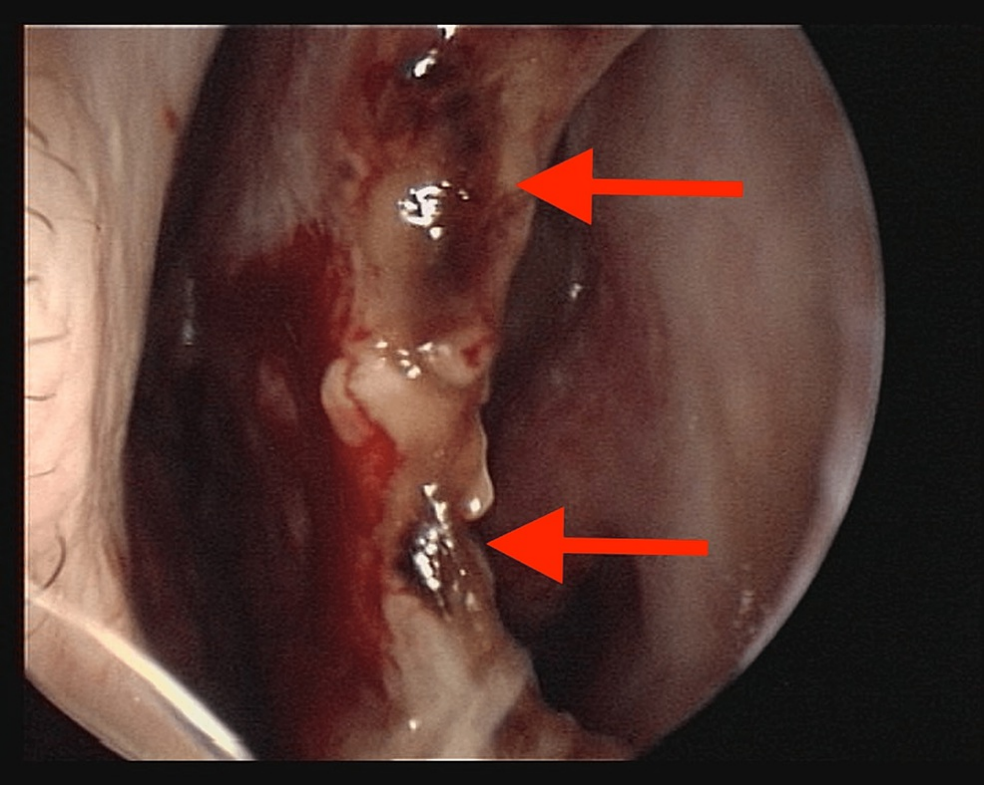
Nasal septum perforation Perforation of the nasal septum seen at rhinoscopy with necrotic-appearing tissue (red arrows)

Rhinoscopy examination by otolaryngology showed a large perforation involving the cartilaginous nasal septum surrounded by necrotic mucosa, as can be seen in Figure 1.

Otolaryngologists performed multiple biopsies that showed no infectious, granulomatous, or malignant cause of perforation. They then started the patient on local treatment with lubricant and antibiotic lotions to avoid bacterial infection and improve symptoms.

With all these pathology results, aflibercept was withdrawn first. We considered this a non-threatening side effect and because of the high risk of discontinuing antineoplastic treatment in this patient, we reintroduced aflibercept in combination with FOLFIRI. The patient gradually improved the local symptoms without complete resolution of the perforation during otolaryngology rhinoscopy monitoring. However, after 16 months, follow-up was discontinued due to disease progression and death.

## Discussion

Nasal septum perforation is a clinical entity usually related to medical fields such as traumatology, infectious diseases, and substance abuse [6]. However, in cancer patients, it is an atypical entity. In this population, this AE is most seen with antiangiogenic treatment, particularly with bevacizumab [4-5].

A literature search of PubMed found few case reports published of septum perforation with bevacizumab [7-16]. The scientific literature regarding the incidence of this complication is scarce. One study found that five subjects (7%) out of 70 metastatic breast cancer patients treated with bevacizumab developed nasal septum perforation [5]. Another study on metastatic colorectal patients found that one subject (1%) out of 100 metastatic colorectal cancer patients developed this AE [4].

In addition, in one article about two cases, we found evidence that docetaxel may also be associated with nasal septal perforation [17].

The management of this entity is not clearly defined. It remains mainly symptomatic in most cases. Treatment is usually based on local measures, intranasal hydration, lubricant, antibiotic lotions, and hemostatic agents in the event of epistaxis. Surgical intervention is seldom needed, as this complication is usually controlled and successfully treated with local measures, especially in patients with poor prognoses [4-5]. There is no evidence for discontinuing antiangiogenic treatment. Therefore, a case-to-case approach has to be done, balancing the risks of withdrawing versus the benefits of continuing treatment. In our case, we decided to continue therapy with aflibercept due to the sole pelvic implant, excellent performance status, and absence of significant toxicities other than nasal septum necrosis.

Only another case report regarding nasal septum perforation related to aflibercept therapy has been published. The article reports a 58-year-old metastatic colorectal cancer patient with nasal septum perforation treated with FOLFIRI plus aflibercept. The subject had no previous history of nasal pathology. Rhinoscopy evaluation revealed a perforation of the inferior portion of the nasal septum. He was started on topical treatment with resolution of epistaxis. Aflibercept was then restarted with no new episodes of epistaxis or other bleeding complications. Follow-up evaluations showed a persistent lesion with progressive epithelization and resolution [18].

## Conclusions

Nasal septum perforation is a rare complication of antiangiogenic treatment usually associated with bevacizumab. In this case report, we show what, to our knowledge, is the second case report of aflibercept-related nasal septum complication. This case report, along with the previously published one, shows that conservative treatment of the lesion and restarting therapy with aflibercept might be appropriate measures.
